# Monoclonal Antibody Dispensing Around Pregnancy: A Descriptive Analysis Using Electronic Health Records in Italy

**DOI:** 10.1002/pds.70518

**Published:** 2026-11-05

**Authors:** Elisabeth Aiton, Valeria Nazzari, Rosie Cornish, Benjamin G. Faber, Christy Burden, Katherine Birchenall, Maria Carolina Borges, Deborah A. Lawlor, Luisa Zuccolo

**Affiliations:** ^1^ Population Health Sciences, Bristol Medical School University of Bristol Bristol UK; ^2^ MRC Integrative Epidemiology Unit University of Bristol Bristol UK; ^3^ Health Data Science Centre Human Technopole Milan Italy; ^4^ MOX, Department of Mathematics Politecnico di Milano Milan Italy; ^5^ Musculoskeletal Research Unit University of Bristol Bristol UK; ^6^ Translational Health Sciences, Bristol Medical School University of Bristol Bristol UK; ^7^ Department of Obstetrics and Gynaecology St Michael's Hospital Bristol UK; ^8^ School of Medicine and Public Health, Hunter Medical Research Institute University of Newcastle Newcastle Australia

**Keywords:** autoimmune, biologics, descriptive, dispensing, monoclonal antibody, pregnancy, real‐world evidence

## Abstract

**Purpose:**

To describe trends in dispensing of monoclonal antibody biologics (mAbs) for autoimmune conditions during and around pregnancy.

**Methods:**

We conducted a population‐wide study of women of reproductive age (14–49 years) resident in Lombardy, Italy, between 2012 and 2024. We described trends over time in the prevalence of mAb dispensations among women of reproductive age, and during pregnancy. For each pregnancy, we assessed the prevalence of mAb dispensations before, during, and after pregnancy in trimesters. We described dispensing patterns and explored maternal factors associated with discontinuation.

**Results:**

We included 1 871 929 women of reproductive age and 855 865 pregnancies. Prevalence of mAb dispensing during pregnancy increased over 60‐fold over the study period, from 0.0041% in 2012 to 0.27% in 2024. Pregnancy affected mAb dispensing, with prevalence decreasing from 0.081% before pregnancy to 0.051% by the third trimester. Over half (*n* = 385/722, 53.3%) of pre‐existing mAb users discontinued before or during pregnancy, while switching between mAbs during pregnancy was rare (*n* = 24/722, 3.3%). Discontinuation decreased over time, and varied substantially between mAbs. We found limited evidence that sociodemographic factors were associated with discontinuation, but that some health factors may be, such as use of assisted reproductive technology (OR = 1.92, 95% CI: 0.98–3.77).

**Conclusions:**

Italian population‐wide data from 2012 to 2024 show an increase in mAbs dispensed during pregnancy, and fewer instances of discontinuing these drugs over time. These trends may reflect increasing drug availability, accumulating safety evidence, and recent changes in guidelines for mAb use in pregnancy.

## Key Points

- We found that the use of monoclonal antibodies in women of reproductive age and during pregnancy steadily increased in Lombardy between 2012 and 2024.
- The most dispensed monoclonal antibody in 2024 was adalimumab for women of reproductive age, while during pregnancy it was certolizumab pegol.
- Around half of women who were dispensed monoclonal antibodies prior to pregnancy discontinued just before or during their pregnancy.
- Discontinuation varied between drugs; it was less likely for TNF inhibitors and more likely for drugs most recently approved by the European Medicines Agency.
- We found that discontinuation decreased over time, in line with accumulating observational safety data and updated subspecialty guidelines.

## Plain Language Summary

People with autoimmune conditions who experience moderate or severe symptoms are often treated using monoclonal antibody drugs, which may also be called biologics. There are some concerns around whether these drugs are safe to use during pregnancy because there is limited and emerging data on how they affect maternal and neonatal complications. We studied women of reproductive age in Lombardy, Italy, between 2012 and 2024. Our aim was to describe trends in how monoclonal antibodies are dispensed around pregnancy. We found that over this period the use of these drugs increased substantially in women of reproductive age and during pregnancy. Around half of women stopped using monoclonal antibodies just before or during their pregnancy, though this varied between drugs. Stopping during pregnancy was also more likely if a pregnancy was conceived using fertility treatments such as in vitro fertilisation (IVF). Switching between monoclonal antibodies during pregnancy only occurred in around one in 30 pregnancies. In more recent years, women were more likely to continue using monoclonal antibodies throughout pregnancy. This trend fits with recent recommendations from medical organisations and may be influenced by new safety data, changes in symptom severity among women, or increasing availability of these drugs.

## 1 Introduction

In Europe, autoimmune conditions affect 7%–13% of women, and are frequently diagnosed during reproductive age [1, 2]. Moderate to severe autoimmune conditions are increasingly managed by monoclonal antibody therapeutics (mAbs) in high‐income countries [3, 4, 5]. A recent Italian study found that 10.5% of women with psoriasis and 20.1% of women with rheumatoid arthritis used mAbs prior to pregnancy [6].

mAbs provide a targeted and effective treatment due to their immunoglobulin G structure, but this structure also means most are actively transported across the placenta from the second trimester as fetal passive immunity develops [7, 8, 9, 10]. This has led to safety concerns around impacts on the neonate's immune system. Clinical guidelines have therefore often advised discontinuing treatment by the second trimester where possible [11, 12, 13, 14, 15]. The latest updates from the European Alliance of Associations for Rheumatology [16] and the European Crohn's and Colitis Organization [17] recommended the use of TNF inhibitor mAbs throughout pregnancy where needed to manage active disease, based on growing reassuring evidence from observational studies [18]. For other mAbs, recommendations were more cautious due to sparse data [16]. The decision to discontinue mAb treatment during pregnancy requires women and clinicians to weigh the risks posed by recurrence of active disease, since active autoimmune conditions may increase risks of adverse pregnancy outcomes [19, 20, 21], against the often limited evidence on mAbs' safety profiles [8]. The limited evidence often causes conflicting advice on whether and when to discontinue in pregnancy.

Limited data describe current dispensing patterns during pregnancy for most mAbs. Some studies have described decreases in mAb dispensing during pregnancy [6, 22, 23, 24, 25, 26, 27], though all literature in this area is limited by modest sample sizes [6, 23], and studies have often focussed on only one autoimmune condition [4, 6, 24] or only on TNF inhibitors [26]. To our knowledge, no previous studies have described trends in mAb dispensing over time among reproductive age women. We aim to address this gap and describe dispensing patterns around pregnancy in recent population‐wide data from Lombardy. It is important to monitor these patterns to understand current clinical practice, particularly given increasing mAb use and the changing landscape of evidence and clinical guidelines.

We aimed to describe: (1) temporal trends in mAb dispensing in women of reproductive age and during pregnancy, (2) treatment patterns of mAb dispensation before, during and after pregnancy, (3) prevalence of mAb discontinuation, switching between mAbs, and resumption among pre‐pregnancy users, and (4) factors associated with discontinuation of mAbs during pregnancy.

## 2 Methods

### 2.1 Data Source

We used electronic health record data from Lombardy, the largest and most industrialised of Italian regions with a population of approximately 10 million spanning both rural and metropolitan areas. We used an existing cohort of women resident in Lombardy on 01.12.2019 aged 14–49. Data covered the period from 01.01.2012 to 31.12.2024, and included linked demographic information, in‐patient hospital admissions, delivery records (covering pregnancies reaching 20 weeks' gestation), out‐patient and emergency room hospital admissions, drugs dispensed in hospitals or pharmacies and reimbursed by the Italian National Health Service, and reasons for exemptions from dispensation payments. These data capture all residents covered by the National Health Service who accessed services delivered by either public or accredited private healthcare facilities. We capture all deliveries in the region, and all in‐hospital dispensations (class H, including mAbs) [28]. Only out‐patient visits submitted for reimbursement are captured, so we may miss visits covered by private insurance [29]. Table S1 outlines all key variables and their data sources.

### 2.2 Study Population

We used different analysis subsamples of this cohort for our aims, illustrated in Figure S1. Prevalence of mAb dispensations was estimated for all women of reproductive age (aim 1), and among all pregnancies (aims 1 and 2). All pregnancies to women who received a mAb dispensation in the year before pregnancy, during pregnancy, or the year after pregnancy were used to identify treatment patterns (aim 2). Finally, we defined women as ‘pre‐existing users’ if they had received a mAb dispensation in the period between 1 year before and 90 days before pregnancy (aims 3 and 4).

### 2.3 Monoclonal Antibody Dispensing

We previously [21] identified a list of autoimmune conditions which may be diagnosed in women of reproductive age [1]. We searched DrugBank [30] in order to identify mAbs which are used to manage these autoimmune conditions. Full details of the search strategy are outlined in Supporting Information: methods [1, 21, 30, 31] and Figure S2.

We identified 23 mAbs (Table S2) used to treat seven autoimmune conditions: inflammatory bowel disease, rheumatoid arthritis, ankylosing spondylitis, psoriasis, multiple sclerosis, systemic lupus erythematosus, and myasthenia gravis. Relationships between indications, mAbs and their targets are summarised in Figure S3.

For each mAb dispensation, the start date and duration (in days) covered by the dispensation was obtained directly from the hospital dispensing data (see Table S1).

### 2.4 Exposure Periods

For each pregnancy, conception date was estimated as date of delivery minus gestational age at birth in weeks (see Table S1). We then calculated dates for each trimester and approximately 90‐day windows in the year before conception and year after delivery [32], which we refer to as ‘periods’ for simplicity. Definitions for these periods are given in Table S3. We used the mAb dispensation start date and duration to identify dispensations overlapping with a given period. For details of dispensation data cleaning see Supporting information: methods.

### 2.5 Autoimmune Conditions

Autoimmune condition diagnosis was derived from any of: (1) outpatient hospital admissions, (2) emergency room admissions, or (3) reasons for payment exemptions. From admissions records we used diagnoses given as either primary reason for admission or secondary diagnoses. ICD‐9 codes and exemption reasons used are described in Table S4. We were unable to capture all autoimmune condition diagnoses in the population as the hospital dispensing data do not include diagnoses and primary care data were not available.

### 2.6 Statistical Analyses

All statistical analyses were conducted in R (4.3.0). We first calculated the prevalence of receiving a mAb dispensation among women of reproductive age in each calendar year, for any mAb and each mAb individually. Since the age distribution of the study cohort changed slightly over the study period (see Figure S4), we additionally performed this analysis stratified by 5‐year age categories. We then estimated the prevalence of receiving a mAb dispensation during pregnancy for each calendar year of delivery, similarly for any mAb and each individually. We additionally described the prevalence of dispensations across periods or trimesters across the year before, during, and the year after pregnancy.

To describe treatment patterns, we assigned each period in each pregnancy to a mAb or none, using the mAb with the longest dispensation duration in cases of multiple mAb dispensations occuring within the same period.

We then described the prevalence of mAb dispensing patterns around pregnancy, among pre‐existing users. We defined dispensing patterns using the pattern of mAb exposure(s) assigned to each period. We defined discontinuation as at least one dispensation in the period between 1 year before and 90 days before pregnancy, followed by any period without mAb exposure between the 90 days pre‐pregnancy and the third trimester inclusive. The timing of discontinuation was the first of these periods during which the individual received no mAb dispensation. Switching between mAbs was defined as changing the mAb assigned to a period, for any period between the 90 days pre‐pregnancy and the third trimester inclusive. Resumption after pregnancy was defined as discontinuation followed by using any mAb in at least one post‐pregnancy period. We compared dispensing patterns stratified by drug used in the year before pregnancy, for drugs used prior to 20 or more pregnancies.

To investigate sociodemographic differences in these trends, we repeated analyses stratified by maternal factors related to differential comorbidities and access to health services. These were: maternal age at delivery, country of birth, maternal education as a proxy for socioeconomic position [33], and calendar year (Table S1).

We used logistic regression adjusting for calendar year of pregnancy only, to describe the associations of mAbs discontinuation with factors including: country of birth (Italy/other), age at delivery, education, employment status, use of assisted reproductive technology, parity (nulliparous/multiparous), previous miscarriage, any autoimmune condition, drug dispensed before pregnancy, and European Medicines Agency (EMA) approval year for drug dispensed before pregnancy (Table S1). We used minimally adjusted models because we aimed to describe overall associations, rather than estimate confounder‐adjusted causal effects. Each model was restricted to complete cases and clustered by pregnant individual to account for multiple pregnancies [34]. We had planned to investigate dispensing centre as a factor but this was not feasible since most dispensations originated from a single centre. We also planned to describe the association of the same factors with switching between mAbs during pregnancy, but too few women switched between mAbs for this analysis to be included.

## 3 Results

We identified 1 871 929 eligible women of reproductive age. Of these women, 1 504 414 (80%) were resident in Lombardy and of reproductive age for 10 or more years, and 1 089 405 (58%) for the full study period (Table S5). These women had 855 865 pregnancies between 2012 and 2024 in the Lombardy region (Table 1). Mean age at delivery was 32.7 years (standard deviation = 5.2). We identified 164 549 mAb dispensations in the study period.

**TABLE 1:** Characteristics of pregnancies in Lombardy cohort in the study period 2012–2024, stratified by those dispensed mAbs during pregnancy, in the year before pregnancy, and/or in the year post‐partum (‘Exposed’) and not (‘Unexposed’).

	Dispensed mAbs during or around pregnancy (*N* = 1102)	Not dispensed mAbs during or around pregnancy (*N* = 854 763)	Overall (*N* = 855 865)
Delivery year			
Mean (SD)	2020 (3.25)	2020 (3.63)	2020 (3.63)
2012–14	81 (7.4%)	220 048 (25.7%)	220 129 (25.7%)
2015–17	156 (14.2%)	219 066 (25.6%)	219 222 (25.6%)
2018–20	267 (24.2%)	200 949 (23.5%)	201 216 (23.5%)
2021–24	598 (54.3%)	214 700 (25.1%)	215 298 (25.2%)
Maternal age at delivery			
Mean (SD)	33.4 (4.78)	32.7 (5.16)	32.7 (5.16)
≤ 30	290 (26.3%)	273 270 (32.0%)	274 010 (32.0%)
31–33	250 (22.7%)	185 232 (21.7%)	185 482 (21.7%)
34–36	269 (24.4%)	185 311 (21.7%)	185 580 (21.7%)
≥ 37	293 (26.6%)	210 500 (24.6%)	210 793 (24.6%)
Parity			
Multiparous	463 (42.0%)	423 256 (49.5%)	423 719 (49.5%)
Nulliparous	176 (16.0%)	121 132 (14.2%)	121 308 (14.2%)
Missing	463 (42.0%)	310 375 (36.3%)	310 838 (36.3%)
Country of birth			
Italy	913 (82.8%)	612 398 (71.6%)	613 311 (71.7%)
Not Italy	189 (17.2%)	242 343 (28.4%)	242 532 (28.3%)
Missing	0 (0.0%)	22 (0.0%)	22 (0.0%)
Education			
Secondary school or below	194 (17.6%)	186 814 (21.9%)	187 008 (21.9%)
High school	472 (42.8%)	363 794 (42.5%)	364 266 (42.5%)
Undergraduate	61 (5.5%)	40 912 (4.8%)	40 973 (4.8%)
Postgraduate	375 (34.0%)	261 116 (30.5%)	261 491 (30.6%)
Missing	0 (0.0%)	2127 (0.2%)	2127 (0.2%)
Employment status			
Employed	868 (78.8%)	586 860 (68.7%)	588 713 (68.6%)
Unemployed	86 (7.8%)	71 468 (8.4%)	71 554 (8.4%)
Stay at home parent	135 (12.3%)	185 163 (21.7%)	185 298 (21.7%)
Other	12 (1.1%)	9040 (1.1%)	9052 (1.1%)
Missing	1 (0.1%)	2232 (0.3%)	2233 (0.3%)
Autoimmune condition^a^			
Any	184 (16.7%)	4559 (0.5%)	4743 (0.6%)
Inflammatory bowel disease	125 (11.3%)	1447 (0.2%)	1572 (0.2%)
Rheumatoid arthritis	14 (1.3%)	50 (0.0%)	64 (0.0%)
Psoriasis	25 (2.3%)	2385 (0.3%)	2410 (0.3%)
Multiple sclerosis	15 (1.4%)	490 (0.1%)	505 (0.1%)

*Note:* Autoimmune condition diagnoses were derived from secondary care and/or dispensation payment exemption records, and are not complete since these diagnoses were not included in hospital dispensation data and primary care records were not available.

^a^ Codelists provided in Table S4.

Among all pregnancies, 1102 (0.13%) received a mAb dispensation in the year before pregnancy, during pregnancy, and/or in the year after pregnancy. The duration of mAb dispensations overlapping with pregnancy (median 57 days, interquartile range = 29–114) was similar to dispensations which did not overlap with pregnancy (median 60 days, interquartile range = 30–110; Table S6).

Pregnancies exposed to mAbs were more likely to be to mothers who had an autoimmune condition diagnosis recorded in secondary care or dispensation payment exemptions (16.7% vs. 0.5%), which were largely records of inflammatory bowel disease (11.3% vs. 0.2%) (Table 1). They were also more likely to be born in 2021–2024 (54.3% vs. 25.1%), and to mothers who were born in Italy (82.8% vs. 71.6%), had postgraduate‐level education (34.0% vs. 30.5%), and were employed (78.8% vs. 68.7%).

### 3.1 Temporal Trends in Dispensing

Among women of reproductive age, the prevalence of receiving a mAb dispensation increased from 0.049% in 2012 to 0.42% in 2024 (Table S7). This trend was consistent across all mAbs (Figure 1) and age groups (Figures S5 and S6). Adalimumab was the most dispensed mAb overall, dispensed to 0.10% of reproductive age women in 2024.

**FIGURE 1:**
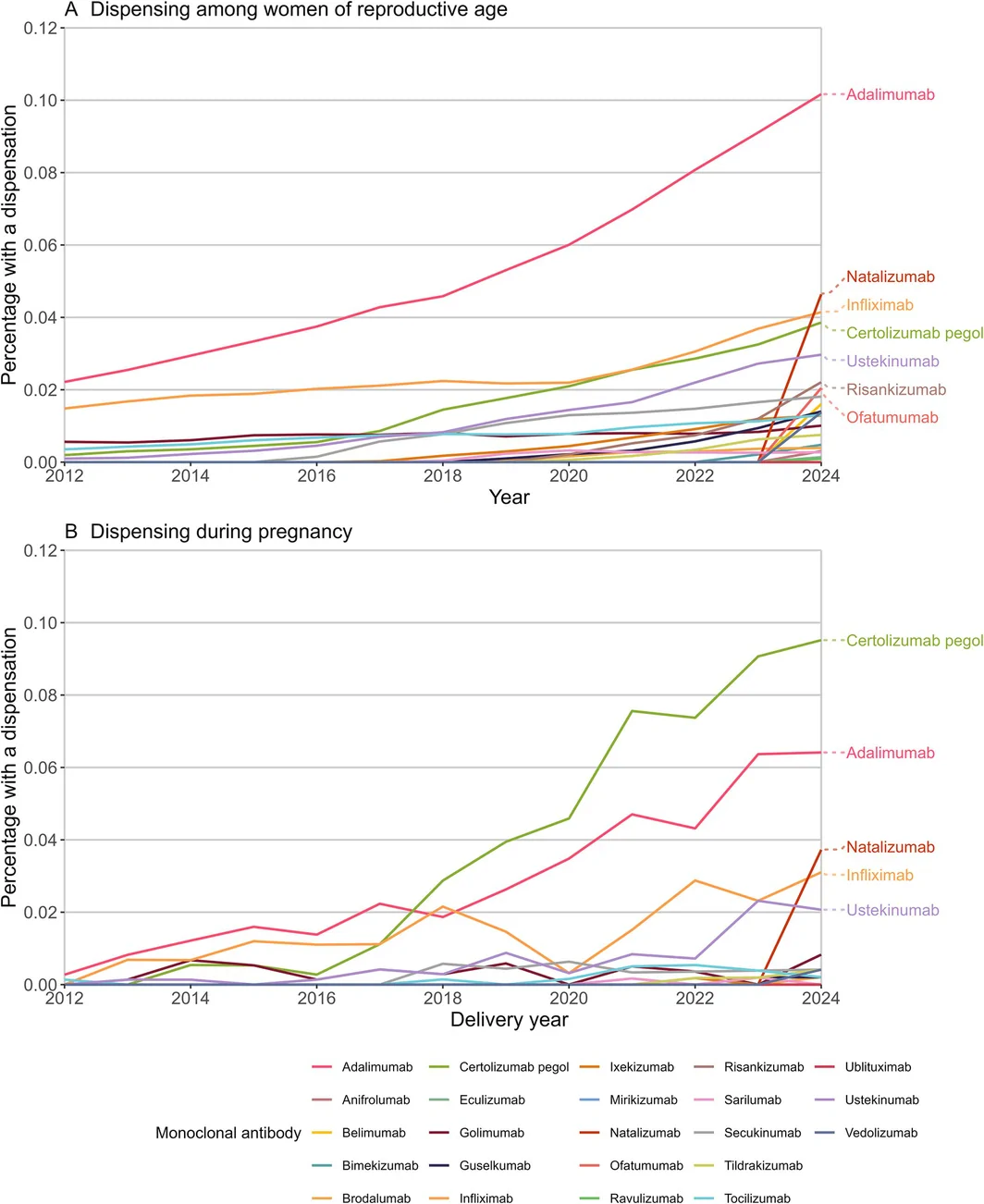
Trends in prevalence of monoclonal antibody dispensations over time in Lombardy. (A) Trend in dispensation among all women of reproductive age by calendar year. (B) Trends in dispensation during pregnancy by calendar year of delivery. Drug name refers to the active ingredient, which may be administered as different pharmaceutical products.

During pregnancy, there was also an increase in prevalence of mAb dispensing over the study period from 0.0041% in 2012 to 0.27% in 2024 (Table S8). This trend was consistent for the year before and the year after pregnancy (Figure S7), and similarly across maternal education and age at delivery, but dispensing was consistently slightly higher among women born in Italy (Figure S8). From 2018 onwards, certolizumab pegol was the most dispensed mAb during pregnancy (0.095% in 2024; Figure 1), while adalimumab was the second‐most dispensed (0.064% in 2024). Natalizumab dispensing increased rapidly from 2023 to 0.037% of pregnancies in 2024.

### 3.2 Patterns of Dispensing During and Around Pregnancy

Prevalence of any mAb dispensation decreased during pregnancy, from 0.081% before pregnancy to 0.064% and 0.051% during the second and third trimesters, respectively (Figure S9; Table S9). Prevalence also increased slightly after pregnancy, with dispensing in the period one‐year pre‐pregnancy at 0.071% compared to 0.090% one‐year postpartum. The absolute decrease in dispensing prevalence between the period before pregnancy and the third trimester was greatest for adalimumab (0.026% to 0.012%), infliximab (0.014% to 0.0082%), and ustekinumab (0.0062% to 0.0026%), while only certolizumab pegol was dispensed at a similar prevalence (0.025% to 0.025%) (Figure 2).

**FIGURE 2:**
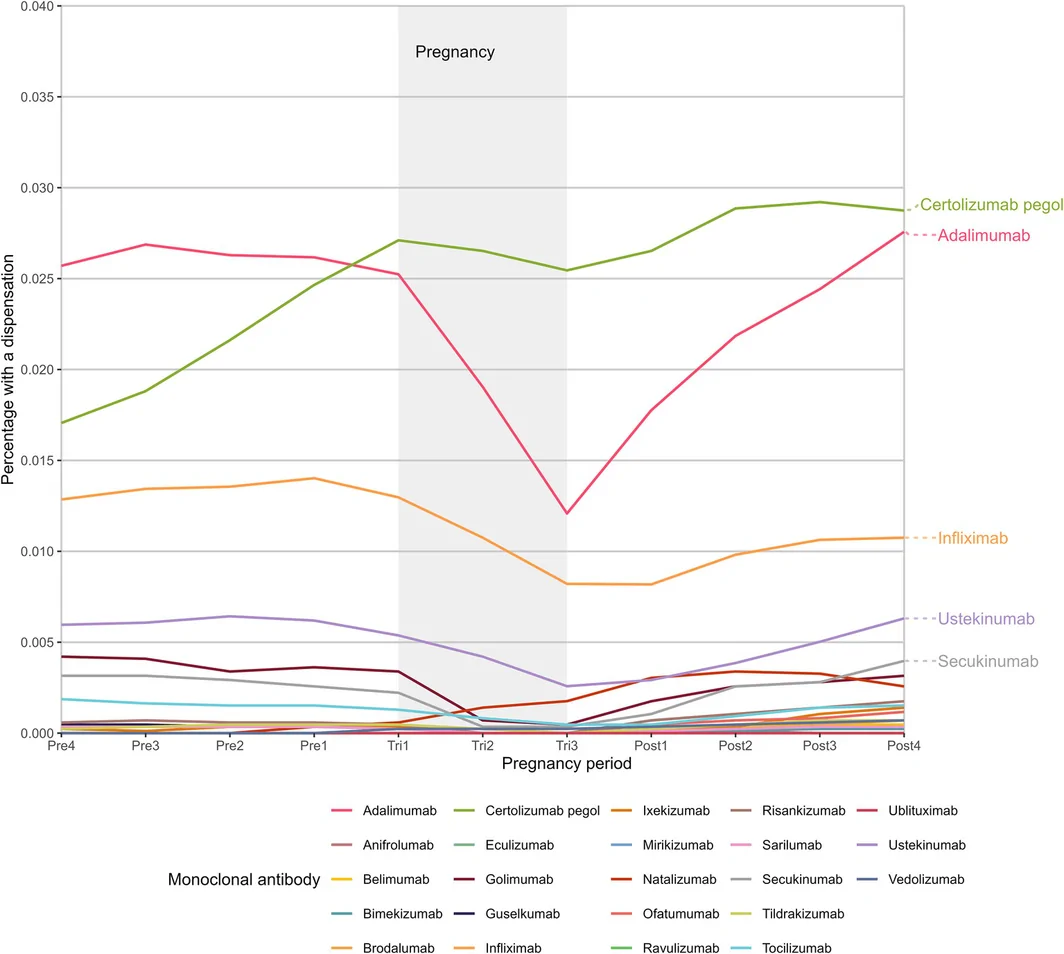
Prevalence of monoclonal antibody dispensation before, during and after pregnancy, stratified by drug. Pregnancy periods are pregnancy trimesters or 90‐day windows before or after pregnancy, for example ‘Pre1’ is the 90 days before conception. Pregnancies losses in the second trimester do not contribute to the denominator for trimester three.

These trends were similar across maternal age at delivery and education (Figure S10). For both more recent delivery years and women born in Italy, overall dispensing prevalence during pregnancy was consistently higher and the decrease in the third trimester was more pronounced.

The treatment patterns of all pregnant women who were exposed to a mAb in one of these periods (*n* = 1102 pregnancies to 948 women; Figure S11), similarly showed that pregnancies were least likely to receive a dispensation overlapping the second (*n* = 547/1102, 49.6%) and third trimesters (*n* = 451/1102, 40.9%). Treatments were largely consistent throughout pregnancy, and very few pregnancies switched between mAbs (Figure S11).

### 3.3 Prevalence of Discontinuation and Switching Between mAbs During Pregnancy and Resumption After Pregnancy

Among women who were exposed to a mAb between 1 year before and 90 days before pregnancy (*n* = 722, ‘pre‐existing users’), the majority discontinued either before or during pregnancy (*n* = 385/722, 53.3%), many continued to receive mAb dispensations during pregnancy (*n* = 337/722, 46.7%), and a small number switched mAb during pregnancy (*n* = 24/722, 3.3%). Of those who discontinued, just over half resumed any mAb treatment in the year post‐delivery (*n* = 215/385, 55.8%).

The most common timing for discontinuation was the second trimester (*n* = 145/385, 37.7%), followed by the third trimester (*n* = 106/385, 27.5%), before pregnancy (*n* = 89/385, 23.1%), and first trimester (*n* = 45/385, 11.7%). Figure 3 summarises overall dispensing patterns (see Table S10 for counts), which were largely consistent across strata of maternal factors. However, continuation of mAb dispensations throughout pregnancy was more prevalent in later delivery years, increasing from 18.2% (*n* = 8/44) in 2012–14 to 58.3% (*n* = 233/400) in 2021–2024. There were also expected differences in dispensing patterns dependent on the drug dispensed in the year before pregnancy (Figure S12, analysis restricted to drugs used prior to 20 or more pregnancies). Certolizumab pegol and multiple mAb users were the most likely to continue the same drug throughout all three trimesters (65.3% (*n* = 113/173) and 50.8% (*n* = 31/61), respectively).

**FIGURE 3:**
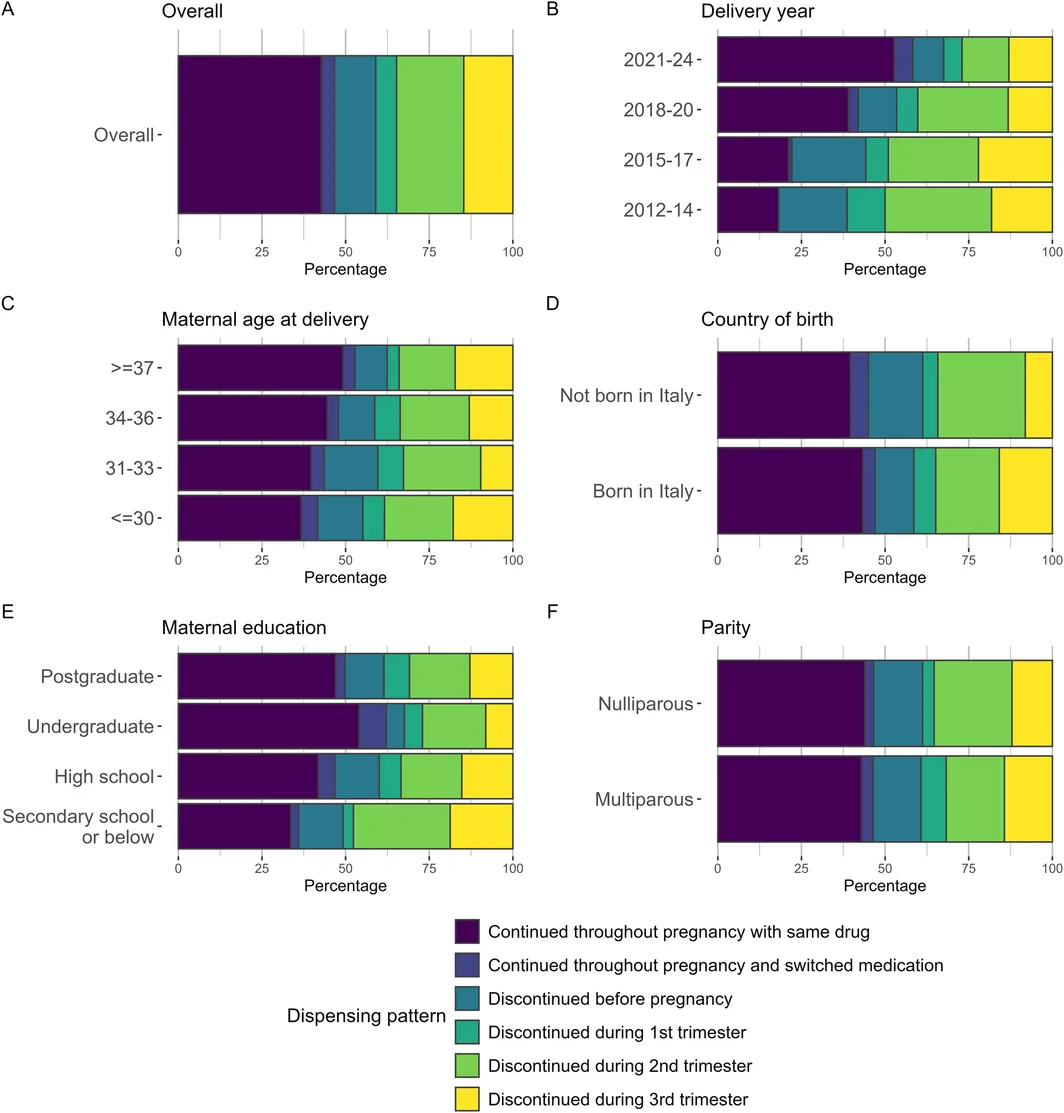
Prevalence of dispensing patterns before and during pregnancy, among pre‐existing monoclonal antibody users (*n* = 722 pregnancies to 630 women), overall (A) and stratified by maternal factors (B–F). Pre‐existing users were defined as exposed to a mAb between 1 year before and 90 days before pregnancy. A small number of pregnancies which discontinued also switched between monoclonal antibodies (*n* < 10). Pregnancies with missing information on maternal factors were discarded, resulting in a reduced sample size for parity only (*n* = 409 pregnancies).

### 3.4 Factors Associated With Discontinuation

Whether the mother was born in Italy, maternal age, education, employment or parity did not appear to influence odds of discontinuation (Figure S13). We found evidence that compared to those who had conceived spontaneously, those who had conceived by assisted reproductive technology were more likely to discontinue (OR = 1.92, 95% CI: 0.98–3.77). We also found evidence that both previous miscarriage (OR = 0.68; 95% CI: 0.44–1.06) and a recorded autoimmune condition (OR = 0.62, 95% CI: 0.41–0.96) may be associated with decreased odds of discontinuation.

Compared to pre‐existing users dispensed adalimumab in the year before pregnancy, women dispensed infliximab (OR = 0.65, 95% CI: 0.41–1.04), certolizumab pegol (OR = 0.51, 95% CI: 0.33–0.81), or multiple mAbs (OR = 0.35, 95% CI: 0.18–0.69) were less likely to discontinue. On the other hand, users of secukinumab (OR = 2.57, 95% CI: 1.04–6.35), ustekinumab (OR = 1.89, 95% CI: 1.01–3.54), and other mAbs (OR = 4.35, 95% CI: 1.86–10.19) were more likely to discontinue. We found evidence that compared to mAbs approved by the European Medicines Agency in 1999 (infliximab), those dispensed mAbs approved in 2003 (adalimumab) and 2015 (secukinumab) were more likely to discontinue (2003 OR = 1.55, 95% CI: 0.97–2.49; 2015 OR = 4.05, 95% CI: 1.56–10.51), while there was more modest evidence for an association for those approved in 2009 (certolizumab pegol, ustekinumab, golimumab; OR = 1.27, 95% CI: 0.80–2.04).

## 4 Discussion

### 4.1 Main Findings

We identified substantial increases in the prevalence of mAb dispensing among both women of reproductive age and pregnant women, between 2012 and 2024 in Lombardy. In 2024, certolizumab pegol was the most widely used mAb during pregnancy, followed by adalimumab and natalizumab. For individual pregnancies, there was a decrease in dispensing from before pregnancy to the third trimester, which recovered post‐partum. Among pre‐existing users, we found one in thirty switched mAbs and around half discontinued before or during pregnancy; among those who discontinued, approximately half went on to receive a mAb dispensation in the year after pregnancy.

The prevalence of discontinuation decreased over time and varied between mAbs. Discontinuation was least common for women who had used certolizumab pegol or multiple mAbs before pregnancy. We found modest evidence that discontinuation was more likely for pregnancies conceived using assisted reproductive technology and negatively associated with a recorded autoimmune condition diagnosis or previous miscarriage, but no evidence for associations with other health or socioeconomic factors.

### 4.2 Interpretation

Few previous studies have estimated the prevalence of mAb use in population‐wide data, and as far as we are aware none have estimated this in women of reproductive age or during pregnancy. Our estimates for the prevalence of mAb dispensations were compatible with previous population‐wide studies of prevalence for both autoimmune conditions [1] and mAbs [5]. In the UK in 2020, the prevalence of mAb use to manage immune‐mediated inflammatory diseases was 0.089% in all people of reproductive age (aged 18–49), and 0.127% among all women [5]. We found comparable estimates in Lombardy in 2020, with a prevalence of mAb dispensations of 0.160% among reproductive‐age women for a slightly wider range of mAbs. In line with our results, a French study of multiple sclerosis management during pregnancy similarly found increased use of natalizumab in recent years [4]. The increase in mAb dispensations may reflect increases in autoimmune condition diagnoses [1, 35], and/or rising use and availability of mAbs [3, 4, 5, 26].

We found that mAbs were discontinued in around half of pregnancies, in accordance with previous studies across Europe and North America [6, 22, 23, 24, 25, 26, 27]. In line with our findings, a previous Italian study found that mAb use declined during pregnancy across subspecialties [22]. Another study of Italian women in the Lazio region found that women with psoriasis all discontinued mAb treatment during pregnancy, as did the majority of women with rheumatoid arthritis [6]. Their findings may differ from ours due to their smaller sample size (< 100 women per condition using mAbs), focus on psoriasis treatments, older dataset (until 2016), and/or regional differences in healthcare provision [22].

We found a modest increase in the prevalence of mAb dispensation between before and after pregnancy; however, a previous study found that the prevalence of mAb use in the year post‐partum was lower than in the year pre‐pregnancy [22]. In the present study, we also found that just over half of women who discontinued mAbs during pregnancy later resumed mAb treatment post‐partum. The increase in mAb dispensation we saw post‐pregnancy is likely to reflect temporal trends in mAb use, since we found that mAb use increased rapidly between 2021 and 2024 and the previous study period ended in 2021 [22]. It might also be explained by post‐pregnancy flare‐ups in disease activity or post‐pregnancy disease onset commonly seen in rheumatoid arthritis and multiple sclerosis [36, 37].

Across strata of maternal age, country of birth and education, we found that trends were broadly consistent for both trends of increased mAb use over time (aim 1) and patterns of dispensation during pregnancy (aim 2). mAb use was higher for women born in Italy than abroad, which could reflect differences in age, autoimmune condition prevalence [38], or differential access to healthcare in Lombardy.

Our finding that discontinuation was common in the second and third trimesters, and varied between drugs, aligns with European and Italian guidelines [14, 15, 16, 17]. These have advised discontinuing most mAbs at this stage due to the risk of placental antibody transfer, unless the benefits of managing disease outweigh the risk of the drug. Certolizumab pegol use pre‐pregnancy was associated with reduced risk of discontinuation. This is in keeping with guidelines which recommend certolizumab pegol use during pregnancy [16, 17] because the molecule does not contain the Fc portion required for active transport across the placenta, though it may still cross via passive diffusion [10, 39].

Switching between mAbs was uncommon over the period 3 months before pregnancy until delivery. As far as we are aware, no prior studies have described switching between mAb treatments during pregnancy. Our findings may reflect minimal switching, or that switching usually occurs earlier than this for planned pregnancies.

mAb discontinuation during pregnancy varied between drugs and decreased between 2012 and 2024. This change has been reported previously for Italy in data up to 2021 [22]. The decrease in discontinuation may reflect updates to guidelines [11, 12, 13, 16], greater clinician familiarity with biologics, and emerging safety data [18, 40]. Safety data is most reassuring for TNF inhibitors [18], and is emerging for natalizumab [41, 42], vedolizumab, and ustekinumab [40]. On the other hand, evidence is still lacking for some more novel and less widely used mAbs [8]. Decreasing mAb discontinuation during pregnancy may also be driven in part by changes in disease severity over time, trends towards older maternal age, increasing availability of biologics, and changing reimbursement policies [43].

We found modest evidence that discontinuation was more likely for pregnancies conceived by assisted reproductive technology and less likely for women with autoimmune diagnoses and previous miscarriage, but unrelated to age or other sociodemographic factors. Similarly, other studies have identified few maternal factors related to mAb discontinuation except drug or calendar year [22, 23, 24]. Our finding for miscarriage is in line with a Canadian study which found some evidence that prior adverse birth outcomes may be associated with lower odds of mAb discontinuation in the first trimester (OR = 0.22, 95% CI: 0.05–0.95) [24].

Our finding that both continuation and discontinuation of mAb treatment were common during pregnancy highlights the challenge of making evidence‐based decisions using limited evidence. Further research is needed into the causal effects of mAbs on the health outcomes of pregnant women and their babies, particularly for more novel therapies [16]. This evidence may be provided by ongoing clinical trials [44] and observational cohort studies. Studies using electronic health record data would require very large sample sizes and linked data across maternity records, hospital medicines, and primary care to account for indications.

### 4.3 Strengths and Limitations

This is the first study to describe trends in mAb dispensations among all women of reproductive age and all pregnancies, and to describe switching between mAbs during pregnancy, as far as the authors are aware. We were able to integrate data on secondary care dispensing and delivery records for a cohort of over 1.8 million women of reproductive age, through to 2024. We are confident that we captured the vast majority of mAbs dispensed in Lombardy since we have access to complete data on hospital‐based drug dispensing, which is the standard approved practice for dispensation of this class of drugs in Italy.

A limitation was the modest sample size of women dispensed mAbs, since mAbs are reserved to treat moderate to severe autoimmune conditions. We did not have complete data on autoimmune condition diagnoses since primary care records were not available and our data only captured publicly reimbursed out‐patient visits, so we could not stratify by or explore the role of specific conditions. However, the vast majority of mAbs studied here are only indicated for autoimmune conditions. The only exceptions were ofatumumab (licensed for chronic lymphocytic leukaemia by the EMA 2010–2019) and tocilizumab (adjunct therapy for Covid‐19 and cytokine release syndrome), to which only 15 and 30 pregnancies were exposed throughout the study period, respectively. Importantly, we also lacked data on disease severity. Disease severity is a key consideration in the decision whether to discontinue mAbs during pregnancy [16, 17], so lacking this information limited our analysis of factors associated with discontinuation.

Our analyses did not consider whether conventional non‐mAb therapies were used to manage autoimmune conditions, which is an important consideration in clinical practice. Instead, we strictly defined switching as between mAbs only, and discontinuation as discontinuing mAbs only. For example, if a pregnant patient in our sample was no longer dispensed infliximab and transitioned to glucocorticosteroid use during pregnancy, we defined this as ‘discontinuing mAbs’. Moreover, dispensing data may not correspond to medication use in practice. Dispensed drugs may not be administered to patients, and dosages might be adjusted during pregnancy. For example, clinicians may not use the full dose in a dispensed vial, or patients may not administer all dispensed pre‐filled syringes stored at home. In these scenarios, the dispensation data would overestimate treatment durations, and our analyses would underestimate the prevalence of discontinuation.

We could only include pregnancies reaching 20 weeks' gestation, therefore excluding early pregnancy losses. If certain autoimmune conditions or mAbs were associated with increased risk of early pregnancy losses, this could introduce selection where individuals with these autoimmune conditions or using these mAbs could be under‐represented in our cohort compared to our target population of all individuals using mAbs who became pregnant [45]. This would result in the prevalence of using this mAb (or mAbs used to manage this condition) during pregnancy being underestimated. Several autoimmune conditions are associated with an increased risk of miscarriage, such as rheumatoid arthritis, psoriasis, and systemic sclerosis [20], but there is more limited evidence linking TNF inhibitor and other widely‐used mAbs with risk of pregnancy loss [18]. The average age of the cohort increased over the study period and most autoimmune conditions are age‐related, which may (in part) explain the trend we found towards increased mAb use over time. Reassuringly, we found consistent trends towards increased mAb use within stratified age bands.

Lombardy is a region of high healthcare quality with higher rates of biologic use than other Italian regions [22], and the Italian National Health Service covers these high‐cost medications. Our findings may not be generalisable to other Italian regions or countries with different healthcare systems or funding models, highlighting the importance of replication in different settings.

## 5 Conclusion

We found increasing mAb use during pregnancy over the past decade in Lombardy, Italy. Over time, mAbs were less likely to be discontinued during pregnancy. Further work is needed to understand the impact of increased mAb use during pregnancy on both maternal and neonatal outcomes, particularly for novel and rarely‐used mAbs.

## Author Contributions

Elisabeth Aiton, Maria Carolina Borges, Deborah A Lawlor, and Luisa Zuccolo conceived and designed the study. Elisabeth Aiton wrote an initial analysis plan, undertook data analyses and wrote the first draft of the manuscript. Luisa Zuccolo established the cohort and was responsible for data oversight and management. Elisabeth Aiton, Valeria Nazzari, Rosie Cornish, Benjamin G Faber, Christy Burden, Katherine Birchenall, Maria Carolina Borges, Deborah A Lawlor, and Luisa Zuccolo provided comments and critical revisions on the analysis plan and drafts of the manuscript. All authors read and approved the final manuscript.

## Funding

This research was funded in whole, or in part, by the Wellcome Trust. This study is supported by the UK Medical Research Council and University of Bristol via the Medical Research Council Integrative Epidemiology Unit at the University of Bristol (MC_UU_00032/5). Elisabeth Aiton is supported by a Wellcome Trust PhD studentship (228276/Z/23/Z). Deborah A Lawlor's contribution to this research is supported by the British Heart Foundation (CH/F/20/90003). Deborah A Lawlor is also supported by the European Union's Horizon Europe Research and Innovation Programme under grant agreement no 101137146. UK participants in Horizon Europe Project STAGE are supported by UKRI grant numbers 10112787 (Beta Technology), 10099041 (University of Bristol) and 10109957 (Imperial College London). Maria Carolina Borges is supported by the Leducq Foundation. Benjamin G Faber is funded by a Wellcome Trust Early Career Award (316390/Z/24/Z). The funders had no role in study design, data collection and analysis, decision to publish, or preparation of the manuscript. The views expressed in this paper are those of the authors and not necessarily any of the funders. For the purpose of Open Access, the author has applied a CC BY public copyright licence to any Author Accepted Manuscript version arising from this submission.

## Ethics Statement

This study was carried out according to the Lombardy Region laws on the use of regional healthcare databases for research activities (D.g.r. X/4893, 09/03/2016; D.g.r. XI/491, 02/08/2018; D.g.r. XI/6387, 16/05/2022.), and in particular on COVID‐19 disease (D.g.r XI/3019, 30/03/2020). The ‘direct and indirect impacts of COVID‐19 on maternal and child health’ project is a collaboration between Human Technopole and the Lombardy Region—General Directorate for Welfare, which approved the data access and use, waiving patient's consent.

## Consent

We confirm that all necessary patient/participant consent has been obtained and the appropriate institutional forms have been archived, and that any patient/participant/sample identifiers included were not known to anyone (e.g., hospital staff, patients or participants themselves) outside the research group so cannot be used to identify individuals.

## Conflicts of Interest

Maria Carolina Borges and Deborah A Lawlor received funding from Novartis for unrelated research. Benjamin G Faber has previously received an honorarium from UCB. The other authors declare no conflicts of interest.

## Supporting information


**Figure S1:** Schematic overview of study aims, analysis subsamples, and data analysis.
**Figure S2:** Selection of monoclonal antibodies used to manage autoimmune conditions occurring at reproductive age.
**Figure S3:** Relationships between autoimmune condition indications, monoclonal antibodies of interest, and their targets.
**Figure S4:** Cohort definition and age profile.
**Figure S5:** Dispensing prevalence of any monoclonal antibody in each calendar year among women of reproductive age, stratified by age band.
**Figure S6:** Dispensing prevalence of each monoclonal antibody in each calendar year among women of reproductive age, stratified by age band.
**Figure S7:** Trends in dispensing of monoclonal antibodies over time, stratified by period of exposure during and around pregnancy.
**Figure S8:** Trends in dispensing of monoclonal antibodies during pregnancy over time, stratified by maternal characteristics.
**Figure S9:** Prevalence of any monoclonal antibody dispensation before, during and after pregnancy.
**Figure S10:** Prevalence of monoclonal antibody dispensing during and around pregnancy by period, stratified by maternal characteristics.
**Figure S11:** Treatment patterns of monoclonal antibody dispensations for each period in the year before, during and after pregnancy, for all pregnancies receiving a monoclonal antibody in at least one period (*n* = 1102 pregnancies, *n* = 948 women).
**Figure S12:** Prevalence of different dispensing patterns before and during pregnancy stratified by drug used in the year before pregnancy, among pre‐existing users (*n* = 719).
**Figure S13:** Associations between maternal factors and odds of discontinuation of monoclonal antibody treatment during pregnancy, among pre‐existing users (*n* = 722).
**Table S1:** Key variables and the data sources.
**Table S2:** Monoclonal antibodies used to manage autoimmune conditions which may be diagnosed in women of reproductive age (*n* = 23 drugs).
**Table S3:** Definitions for time periods of study across the year before pregnancy, during pregnancy, and the year following pregnancy.
**Table S4:** ICD‐9 codelists and dispensation payment exemption codes from Regione Lombardia used to identify autoimmune conditions.
**Table S5:** Extent of cohort attrition and reasons.
**Table S6:** Dispensation durations for all monoclonal antibody dispensations, stratified by whether they overlapped with pregnancy.
**Table S7:** Prevalence of monoclonal antibody dispensations among women of reproductive age, per year.
**Table S8:** Prevalence of monoclonal antibody dispensations among pregnancies, per year.
**Table S9:** Prevalence of monoclonal antibody dispensations in each period before, during and after pregnancy.
**Table S10:** Counts of pregnancies following dispensing patterns, among all pregnant women with a recorded monoclonal antibody dispensation in at least one period before pregnancy (pre‐existing users, *n* = 722).

## Data Availability

The data that support the findings of this study were made available from Lombardy Region. Restrictions apply to the availability of these data, which were used under license for this study. Access to individual‐level Lombardy Region data can be obtained by submitting a project application, and are subject to approval.
