# Supplementary material for: Monoclonal Antibody Dispensing Around Pregnancy: A Descriptive Analysis Using Electronic Health Records in Italy
Source: Pharmacoepidemiol Drug Saf. 2026 Nov 5;35(11):e70518. doi: 10.1002/pds.70518 (PMC13740336; doi:10.1002/pds.70518)
Supplement: Supplementary file 1 [file PDS-35-e70518-s001.docx]

Supplementary Material

Table of Contents

[Methods	3](#_Toc239339420)

[Monoclonal antibody selection	3](#_Toc239339421)

[Dispensation data cleaning	3](#_Toc239339422)

[Figures	4](#_Toc239339423)

[Figure S1. Schematic overview of study aims, analysis subsamples, and data analysis.	4](#_Toc239339424)

[Figure S2. Selection of monoclonal antibodies used to manage autoimmune conditions occurring at reproductive age.	5](#_Toc239339425)

[Figure S3. Relationships between autoimmune condition indications, monoclonal antibodies of interest, and their targets.	6](#_Toc239339426)

[Figure S4. Cohort definition and age profile.	7](#_Toc239339427)

[Figure S5. Dispensing prevalence of any monoclonal antibody in each calendar year among women of reproductive age, stratified by age band.	8](#_Toc239339428)

[Figure S6. Dispensing prevalence of each monoclonal antibody in each calendar year among women of reproductive age, stratified by age band.	9](#_Toc239339429)

[Figure S7. Trends in dispensing of monoclonal antibodies over time, stratified by period of exposure during and around pregnancy.	10](#_Toc239339430)

[Figure S8. Trends in dispensing of monoclonal antibodies during pregnancy over time, stratified by maternal characteristics	12](#_Toc239339431)

[Figure S9. Prevalence of any monoclonal antibody dispensation before, during and after pregnancy.	13](#_Toc239339432)

[Figure S10. Prevalence of monoclonal antibody dispensing during and around pregnancy by period, stratified by maternal characteristics.	14](#_Toc239339433)

[Figure S11. Treatment patterns of monoclonal antibody dispensations for each period in the year before, during and after pregnancy, for all pregnancies receiving a monoclonal antibody in at least one period (n=1,102 pregnancies, n=948 women).	15](#_Toc239339434)

[Figure S12. Prevalence of different dispensing patterns before and during pregnancy stratified by drug used in the year before pregnancy, among pre-existing users (n=719).	16](#_Toc239339435)

[Figure S13. Associations between maternal factors and odds of discontinuation of monoclonal antibody treatment during pregnancy, among pre-existing users (n=722).	17](#_Toc239339436)

[Tables	18](#_Toc239339437)

[Table S1. Key variables and the data sources.	18](#_Toc239339438)

[Table S2. Monoclonal antibodies used to manage autoimmune conditions which may be diagnosed in women of reproductive age (n=23 drugs).	20](#_Toc239339439)

[Table S3. Definitions for time periods of study across the year before pregnancy, during pregnancy, and the year following pregnancy.	21](#_Toc239339440)

[Table S4. ICD-9 codelists and dispensation payment exemption codes from Regione Lombardia used to identify autoimmune conditions.	22](#_Toc239339441)

[Table S5. Extent of cohort attrition and reasons.	23](#_Toc239339442)

[Table S6. Dispensation durations for all monoclonal antibody dispensations, stratified by whether they overlapped with pregnancy.	24](#_Toc239339443)

[Table S7. Prevalence of monoclonal antibody dispensations among women of reproductive age, per year.	25](#_Toc239339444)

[Table S8. Prevalence of monoclonal antibody dispensations among pregnancies, per year.	26](#_Toc239339445)

[Table S9. Prevalence of monoclonal antibody dispensations in each period before, during and after pregnancy.	27](#_Toc239339446)

[Table S10. Counts of pregnancies following dispensing patterns, among all pregnant women with a recorded monoclonal antibody dispensation in at least one period before pregnancy (pre-existing users, n=722).	28](#_Toc239339447)

# Methods

## Monoclonal antibody selection

We have previously [21] identified a list of autoimmune conditions which may be diagnosed in women of reproductive age (defined as those aged 14-49 years), on the basis of a study describing sex-stratified autoimmune condition prevalence [1].

We selected mAbs indicated to manage these autoimmune conditions. First, we mapped mAbs listed in the WHO’s Anatomical Therapeutic Chemical classification ‘L04: Immunosuppressants’ to their indications in adults using DrugBank [30] and the European Medicines Agency (EMA) [31]. We then selected only mAbs used to manage autoimmune conditions which can be diagnosed in women of reproductive age.

We additionally required mAbs to be approved for use in the EMA, and recommended for routine courses of treatment more frequently than every 6 months so that we could accurately capture whether dispensing changed during and around pregnancy.

## Dispensation data cleaning

Throughout all analyses, we required a mAb dispensation to overlap with a period to define it as exposed. Among all mAb dispensations in the study period, a small proportion (1.7%) of dispensation durations were longer than 365 days and these were trimmed to 365 days to remove implausible data, since mAb injections may be stored and administered at home but are unlikely to last longer than one year.

# Figures

## Figure S1. Schematic overview of study aims, analysis subsamples, and data analysis.

## Figure S2. Selection of monoclonal antibodies used to manage autoimmune conditions occurring at reproductive age.

## Figure S3. Relationships between autoimmune condition indications, monoclonal antibodies of interest, and their targets.

## Figure S4. Cohort definition and age profile.

Since the cohort was defined as all women of reproductive age resident in Lombardy in 2019, the age profile of the cohort changed slightly over the study period (2012-2024). The blue shaded areas represent eligible age ranges for a given year, while red shaded areas represent ineligible age ranges.

## Figure S5. Dispensing prevalence of any monoclonal antibody in each calendar year among women of reproductive age, stratified by age band.

95% confidence intervals shown around each age band. This analysis was performed since the cohort was defined as all women of reproductive age resident in Lombardy in 2019, so the age profile of the cohort changed slightly over the study period.

## Figure S6. Dispensing prevalence of each monoclonal antibody in each calendar year among women of reproductive age, stratified by age band.

95% confidence intervals shown around each age band. This analysis was performed since the cohort was defined as all women of reproductive age resident in Lombardy in 2019, so the age profile of the cohort changed slightly over the study period.

## Figure S7. Trends in dispensing of monoclonal antibodies over time, stratified by period of exposure during and around pregnancy.

Categories are mutually inclusive, i.e. one pregnancy could be classed as dispensed monoclonal antibodies in the year prior, during and/or after pregnancy. Bands illustrate 95% confidence intervals around annual prevalence estimates.

Figure S8. Trends in dispensing of monoclonal antibodies during pregnancy over time, stratified by maternal characteristics.

A) Maternal age, B) Maternal country of birth, C) Maternal education level. Bands illustrate 95% confidence intervals around annual prevalence estimates.

## Figure S9. Prevalence of any monoclonal antibody dispensation before, during and after pregnancy.

Pregnancy periods are pregnancy trimesters or 90-day windows before or after pregnancy, for example ‘Pre1’ is the 90 days before conception. Pregnancies losses in the second trimester do not contribute to the denominator for trimester three.

## Figure S10. Prevalence of monoclonal antibody dispensing during and around pregnancy by period, stratified by maternal characteristics.

A) Maternal age, B) Maternal country of birth, C) Maternal education level. Bands illustrate 95% confidence intervals around annual prevalence estimates, accounting for multiple pregnancies to the same individual. Denominator is all pregnancies in the study period (2012-2024).

## Figure S11. Treatment patterns of monoclonal antibody dispensations for each period in the year before, during and after pregnancy, for all pregnancies receiving a monoclonal antibody in at least one period (n=1,102 pregnancies, n=948 women).

The size of the bar at each period is proportional to the count of pregnancies receiving that monoclonal antibody (mAb) or none, for all uncensored pregnancies. For periods with multiple mAb dispensations the dispensation with the longest duration in that period was selected (i.e. if someone received adalimumab for 8 days and certolizumab pegol for 27 in one period, the period would be designated as exposed to certolizumab pegol). Treatment patterns containing fewer than 10 pregnancies have been censored. The ‘Other’ category includes mAbs used in 10 or fewer pregnancies: tocilizumab, risankizumab, guselkumab, ixekizumab, ofatumumab, brodalumab, tildrakizumab, vedolizumab, belimumab, sarilumab, bimekizumab, and anifrolumab.

## Figure S12. Prevalence of different dispensing patterns before and during pregnancy stratified by drug used in the year before pregnancy, among pre-existing users (n=719).

Pre-existing users defined as exposed to a monoclonal antibody in at least one period between 1 year before and 90 days before pregnancy. Drugs used by fewer than 20 pregnancies were discarded in this analysis, resulting in a reduced sample size for this analysis (n=684/719 pre-existing users in sample).

## Figure S13. Associations between maternal factors and odds of discontinuation of monoclonal antibody treatment during pregnancy, among pre-existing users (n=722).

All estimates were adjusted for the effect of calendar year only, and included complete records for each factor. Pre-existing user defined as exposed to a monoclonal antibody between 1 year before and 90 days before pregnancy. ART = assistive reproductive technology. EMA = European Medicines Agency. Autoimmune condition diagnoses were derived from secondary care and/or dispensation payment exemption records only.

# Tables

## Table S1. Key variables and the data sources.

These variables were used to describe dispensing patterns, stratify analyses and/or explored as factors which may be associated with discontinuation. ART = assisted reproductive technology. EMA = European Medicines Agency. See Table S4 for codelists used to define each autoimmune condition.

| **Variable** | **Data source** | **Coding** |
| --- | --- | --- |
| Dispensation date | Hospital drug dispensation data | Date |
| Dispensation duration | Hospital drug dispensation data | Continuous integer:  days |
| Date of delivery | Delivery records, given as week of the year so assumed to be on Wednesday | Date |
| Gestational age at birth | Delivery records | Continuous integer:  Weeks |
| Pregnancy period | Calculated as date of delivery minus gestational age at birth | Time period |
| Maternal age at delivery | Calculated from date of delivery (delivery records) and maternal birth year (demographic data) | Categoric:  <=30, 31-33, 34-36, >=37.  Continuous integer:  years |
| Country of birth | Demographic data | Categoric:  Born in Italy, Not born in Italy. |
| Maternal education | Delivery records | Categoric:  Secondary school or below, High school, Undergraduate, Postgraduate. |
| Calendar year of delivery | Delivery records | Categoric:  2012-14, 2015-17, 2018-20, 2021-24.  Continuous:  years |
| Use of ART | Delivery records | Binary:  Yes, no. |
| Drug dispensed in year before pregnancy | Hospital drug dispensation data, covering the year before pregnancy started. | Categoric:  Adalimumab, Infliximab, Certolizumab pegol, Golimumab, Multiple drugs, Secukinumab, Ustekinumab, Other (for drugs used before <20 pregnancies). |
| Drug EMA approval year | Drug obtained from ‘Drug dispensed in year before pregnancy’ variable. EMA for approval year (retrieved 20/02/2026 (4)). | Categoric:  1999 (Infliximab), 2003 (Adalimumab), 2009 (Certolizumab pegol, Ustekinumab, Golimumab), 2015 (Secukinumab). |
| Employment status | Delivery records | Categoric:  Employed, Stay at home parent, Unemployed or other (Unemployed, Looking for first job, Student or Other (retired, unable)). |
| Autoimmune condition | Outpatient hospital admissions, emergency room admissions, reasons for payment exemptions. | Binary:  Any recorded diagnosis prior to pregnancy or any payment exemption, or none.  Categoric:  Inflammatory bowel disease, Rheumatoid arthritis, Ankylosing spondylitis, Psoriasis, Multiple sclerosis, Systemic lupus erythematosus, Myasthenia gravis. |
| Parity | Delivery records | Binary:  Nulliparous, multiparous. |
| Previous miscarriage | Delivery records | Binary:  Yes, no. |

## Table S2. Monoclonal antibodies used to manage autoimmune conditions which may be diagnosed in women of reproductive age (n=23 drugs).

ATC = Anatomical Therapeutic Chemical classification system.

| **Drug name** | **ATC code** | **Target** | **Autoimmune indications** |
| --- | --- | --- | --- |
| Ravulizumab | L04AJ02 | C5 | Myasthenia gravis |
| Eculizumab | L04AJ01 | C5 | Myasthenia gravis |
| Ublituximab | L04AG14 | MS4A1 | Multiple sclerosis |
| Ofatumumab | L04AG12 | MS4A1 | Multiple sclerosis |
| Anifrolumab | L04AG11 | IFNAR1 | Systemic lupus erythematosus |
| Vedolizumab | L04AG05 | ITGA4; ITGB7 | Inflammatory bowel disease |
| Belimumab | L04AG04 | TNFSF13B | Systemic lupus erythematosus |
| Natalizumab | L04AG03 | ITGA4 | Multiple sclerosis; Inflammatory bowel disease (Crohn's disease) |
| Mirikizumab | L04AC24 | IL23A | Inflammatory bowel disease |
| Bimekizumab | L04AC21 | IL17A; IL17F | Psoriasis; Ankylosing spondylitis |
| Risankizumab | L04AC18 | IL12B; IL23A | Psoriasis; Inflammatory bowel disease |
| Tildrakizumab | L04AC17 | IL23A | Psoriasis |
| Guselkumab | L04AC16 | IL23A | Psoriasis |
| Sarilumab | L04AC14 | IL6R | Rheumatoid arthritis |
| Ixekizumab | L04AC13 | IL17A | Psoriasis; Ankylosing spondylitis |
| Brodalumab | L04AC12 | IL17RA | Psoriasis |
| Secukinumab | L04AC10 | IL17A | Psoriasis |
| Tocilizumab | L04AC07 | IL6R | Rheumatoid arthritis |
| Ustekinumab | L04AC05 | IL12B | Psoriasis; Inflammatory bowel disease |
| Golimumab | L04AB06 | TNF | Rheumatoid arthritis; Psoriasis (psoriatic arthritis) ; Ankylosing spondylitis; Inflammatory bowel disease |
| Certolizumab pegol | L04AB05 | TNF | Rheumatoid arthritis; Psoriasis (psoriatic arthritis) ; Ankylosing spondylitis; Inflammatory bowel disease; Psoriasis |
| Adalimumab | L04AB04 | TNF | Rheumatoid arthritis; Psoriasis (psoriatic arthritis) ; Ankylosing spondylitis; Inflammatory bowel disease; Psoriasis |
| Infliximab | L04AB02 | TNF | Rheumatoid arthritis; Ankylosing spondylitis; Inflammatory bowel disease |

## Table S3. Definitions for time periods of study across the year before pregnancy, during pregnancy, and the year following pregnancy.

| **Period** | **Start (inclusive)** | **End (inclusive)** |
| --- | --- | --- |
| 9-12 months pre-pregnancy | conception - 1 year | conception - 271 days |
| 6-9 months pre-pregnancy | conception - 270 days | conception - 181 days |
| 3-6 months pre-pregnancy | conception - 180 days | conception - 91 days |
| 0-3 months pre-pregnancy | conception - 90 days | conception - 1 days |
| 1st trimester | conception | conception + 90 days |
| 2nd trimester | conception + 91 days | conception + 180 days |
| 3rd trimester | conception + 181 days | delivery |
| 0-3 months postpartum | delivery + 1 day | delivery + 90 days |
| 3-6 months postpartum | delivery + 91 days | delivery + 180 days |
| 6-9 months postpartum | delivery + 181 days | delivery + 270 days |
| 9-12 months postpartum | delivery + 271 days | delivery + 1 year |

## Table S4. ICD-9 codelists and dispensation payment exemption codes from Regione Lombardia used to identify autoimmune conditions.

Diagnoses were obtained from hospital admissions and/or emergency room admissions.

| **Condition** | **ICD-9 codes** | **Payment exemption codes** |
| --- | --- | --- |
| Inflammatory bowel disease | 555*, 556* | 009.555, 009.556 |
| Rheumatoid arthritis | 714 | 006.714.0, 006.714.2 |
| Ankylosing spondylitis | 7200 | 054.720.0 |
| Psoriasis | 6960, 6961, 6968 | 045.696.0, 045.696.1 |
| Multiple sclerosis | 340 | 046.340 |
| Systemic lupus erythematosus | 7100 | 028.710.0 |
| Myasthenia gravis | 358 | 034.558.0, RFG101 |

## Table S5. Extent of cohort attrition and reasons.

Summary of reasons for cohort attrition over the study period 2012-2024. Attrition here is defined as not being eligible in the cohort for one or more years. Attrition may be due to not being of reproductive age (14-49), not being registered in the Lombardy region health system, or both reasons (either in the same or in different years).

| **Attrition from cohort** | **N (%)** |
| --- | --- |
| No attrition | 1,089,405 (58.2) |
| Attrition due to age | 441,473 (23.6) |
| Attrition due to not being registered in Lombardy | 290,386 (15.5) |
| Attrition due to not being registered in Lombardy and due to age | 50,665 (2.70) |

## Table S6. Dispensation durations for all monoclonal antibody dispensations, stratified by whether they overlapped with pregnancy.

| **Dispensation overlap with pregnancy** | **Number of distinct dispensations** | **Median dispensation duration** | **Interquartile range of dispensation duration** | **Mean dispensation duration** |
| --- | --- | --- | --- | --- |
| Not during pregnancy | 161,902 | 60.0 | 30.0 - 110.0 | 81.2 |
| Overlapping with pregnancy | 2,646 | 57.1 | 28.6 - 114.0 | 81.3 |

## Table S7. Prevalence of monoclonal antibody dispensations among women of reproductive age, per year.

| **Year** | **Number of women dispensed monoclonal antibodys** | **Number of monoclonal antibody dispensations** | **Total number of women of reproductive age** | **Prevalence of women receiving any monoclonal antibody dispensation (%)** |
| --- | --- | --- | --- | --- |
| 2012 | 655 | 3539 | 1336031 | 0.049 |
| 2013 | 787 | 4356 | 1401579 | 0.056 |
| 2014 | 948 | 5241 | 1468445 | 0.065 |
| 2015 | 1127 | 6102 | 1537138 | 0.073 |
| 2016 | 1347 | 6958 | 1609503 | 0.084 |
| 2017 | 1691 | 8449 | 1684276 | 0.100 |
| 2018 | 2063 | 10325 | 1767064 | 0.117 |
| 2019 | 2556 | 12440 | 1871929 | 0.137 |
| 2020 | 2926 | 13599 | 1826485 | 0.160 |
| 2021 | 3461 | 15977 | 1811241 | 0.191 |
| 2022 | 4069 | 18663 | 1796786 | 0.226 |
| 2023 | 4624 | 21193 | 1700655 | 0.272 |
| 2024 | 6791 | 30973 | 1607129 | 0.423 |

## Table S8. Prevalence of monoclonal antibody dispensations among pregnancies, per year.

| **Year** | **Number of pregnancies dispensed monoclonal antibodies** | **Total number of pregnancies** | **Prevalence of pregnancies receiving any monoclonal antibody dispensation (%)** |
| --- | --- | --- | --- |
| 2012 | 22 | 73092 | 0.030 |
| 2013 | 22 | 72973 | 0.030 |
| 2014 | 39 | 74064 | 0.053 |
| 2015 | 47 | 75161 | 0.063 |
| 2016 | 47 | 72439 | 0.065 |
| 2017 | 60 | 71622 | 0.084 |
| 2018 | 89 | 69599 | 0.128 |
| 2019 | 85 | 68433 | 0.124 |
| 2020 | 93 | 63184 | 0.147 |
| 2021 | 131 | 59527 | 0.220 |
| 2022 | 125 | 55613 | 0.225 |
| 2023 | 178 | 51828 | 0.343 |
| 2024 | 164 | 48330 | 0.339 |

## Table S9. Prevalence of monoclonal antibody dispensations in each period before, during and after pregnancy.

Individuals may contribute data on more than one pregnancy, so numbers of unique individuals is also presented.

| **Period** | **Number of pregnancies dispensed monoclonal antibodies** | **Number of individuals dispensed monoclonal antibodies** | **Total number of pregnancies** | **Total number of individuals** | **Prevalence of pregnancies receiving any monoclonal antibody dispensation (%)** |
| --- | --- | --- | --- | --- | --- |
| Pre1 | 689 | 604 | 855865 | 599401 | 0.081 |
| Pre2 | 657 | 578 | 855865 | 599401 | 0.077 |
| Pre3 | 636 | 560 | 855865 | 599401 | 0.074 |
| Pre4 | 608 | 536 | 855865 | 599401 | 0.071 |
| Tri1 | 674 | 589 | 855865 | 599401 | 0.079 |
| Tri2 | 547 | 485 | 855865 | 599401 | 0.064 |
| Tri3 | 439 | 397 | 852553 | 597768 | 0.051 |
| Post1 | 548 | 476 | 855865 | 599401 | 0.064 |
| Post2 | 655 | 565 | 855865 | 599401 | 0.077 |
| Post3 | 718 | 629 | 855865 | 599401 | 0.084 |
| Post4 | 771 | 674 | 855865 | 599401 | 0.090 |

## Table S10. Counts of pregnancies following dispensing patterns, among all pregnant women with a recorded monoclonal antibody dispensation in at least one period before pregnancy (pre-existing users, n=722).

| **Dispensing pattern** | **Count (%)** |
| --- | --- |
| Continued throughout pregnancy and switched medication | 28 (3.9%) |
| Continued throughout pregnancy with same drug | 309 (42.8%) |
| Discontinued before pregnancy and did not restart | 49 (6.8%) |
| Discontinued before pregnancy and restarted during pregnancy | 20 (2.8%) |
| Discontinued before pregnancy and restarted post delivery | 20 (2.8%) |
| Discontinued during 1^st^ trimester and did not restart | 19 (2.6%) |
| Discontinued during 1^st^ trimester and restarted post delivery | 26 (3.6%) |
| Discontinued during 2^nd^ trimester and did not restart | 48 (6.6%) |
| Discontinued during 2^nd^ trimester and restarted post delivery | 97 (13.4%) |
| Discontinued during 3^rd^ trimester and did not restart | 34 (4.7%) |
| Discontinued during 3^rd^ trimester and restarted post delivery | 72 (10.0%) |
